# Genotyping of the Duffy Blood Group among *Plasmodium knowlesi*-Infected Patients in Malaysia

**DOI:** 10.1371/journal.pone.0108951

**Published:** 2014-09-30

**Authors:** Jeremy Ryan De Silva, Yee Ling Lau, Mun Yik Fong

**Affiliations:** 1 Department of Parasitology, Faculty of Medicine, University of Malaya, Kuala Lumpur, Malaysia; 2 Tropical Disease Research and Education Centre, Faculty of Medicine, University of Malaya, Kuala Lumpur, Malaysia; Agency for Science, Technology and Research - Singapore Immunology Network, Singapore

## Abstract

The Duffy blood group is of major interest in clinical medicine as it plays an important role in *Plasmodium knowlesi* and *Plasmodium vivax* infection. In the present study, the distribution of Duffy blood group genotypes and allelic frequencies among *P. knowlesi* infected patients as well as healthy individuals in Peninsular Malaysia were determined. The blood group of 60 healthy blood donors and 51 *P. knowlesi* malaria patients were genotyped using allele specific polymerase chain reaction (ASP-PCR). The data was analyzed using Fisher's exact test in order to assess the significance of the variables. Our results show a high proportion of the FY*A/FY*A genotype (>85% for both groups) and a high frequency of the FY*A allele (>90% for both groups). The FY*A/FY*A genotype was the most predominant genotype in both infected and healthy blood samples. The genotype frequency did not differ significantly between the donor blood and the malaria patient groups. Also, there was no significant correlation between susceptibility to *P. knowlesi* infection with any Duffy blood genotype.

## Introduction

The Duffy blood group, also known as the Duffy antigen receptor for chemokines (DARC), is a group of polymorphic molecules located on the exterior portion of the red blood cell (RBC) membrane. The Duffy blood group is of particular importance due to the nature of the Duffy antigen being an obligatory receptor for the invasion of the malaria parasite *P. vivax* and *P. knowlesi* into erythrocytes [1]. Besides being a receptor for various chemokines that facilitate chemokine induced pathways in the body, the Duffy blood group also plays a role in transfusion medicine as antibodies against Duffy antigens have been shown to be responsible for several cases of hemolytic transfusion compatibility and hemolytic disease of the newborn (HDN) [2], [3], [4], [5], [6], [7].

The Duffy blood group was initially reported by Cutbush in 1950, where he described the reactivity of an antibody found in a multitransfused hemophiliac male patient who possessed an alloantibody against an antigen, then denoted as Fy^a^. An allelic form of the antigen, Fy^b^, was described a year later [8]. The *FY* is a single copy gene composed of two exons that encode a protein of 336 amino acids [9]. The FY locus is located on chromosome 1 and is characterized by three main alleles, FY*A, FY*B, and FY*B^ES^.

FY*A and FY*B are codominant alleles distinguished by a mutation (125G>A) which gives rise to the Fy^a^ and Fy^b^ antigens [10]. The antigens differ between each other by one amino acid substitution, the replacement of glycine for aspartic acid at residue 42 of the extracellular domain of the antigen (Gly42Asp) [11]. These two alleles confer the common Duffy phenotypes Fy(a+b+), Fy(a+b−) and Fy(a−b+).

The FY*B^ES^ allele differs from the FY*B allele by a substitution from T to C at the GATA box motif of the FY*B promoter (−33 T>C). This mutation results in a disruption at the binding site of the GATA-1 erythroid transcription factor which in turn results in the loss of *FY* expression in the erythroid lineage but does not affect other tissues [12]. Homozygozity of the FY*B^ES^ allele results in the phenotype Fy(a−b−) which has been shown to render RBC resistance to *P. vivax* malarial infection. This phenotype is more prevalent in human populations of African lineage but is quite rare in Caucasian or Asian populations.

Molecular characterization of the *FY* alleles has allowed for the development of Duffy genotyping by PCR-based approaches such as restriction fragment length polymorphism (RFLP) [9] and allele specific PCR (ASP-PCR) [13].

Natural transmission of the monkey malaria parasite *P. knowlesi* to human was first reported in an American man who had returned from central Peninsular Malaysia in 1965. This was followed by a second case report in southern Peninsular Malaysia 5 years later [14]. Since 2004, after the discovery of a large number of infected patients in Borneo Malaysia [15], there has been an increasing number of naturally acquired *P. knowlesi* malaria among humans in several other Asian countries such as Thailand, The Philippines and Singapore. In Peninsular Malaysia, more than 300 human cases have been detected since 2005 [16], [17], [18].

The aim of the present study is to analyze the distribution of the Duffy genotypes and allelic frequencies of *P. knowlesi* infected patients as well as healthy donor samples in Peninsular Malaysia.

## Materials and Methods

### Blood samples and sample collection

Fifty one *P. knowlesi* infected blood samples were collected from patients admitted to the University of Malaya Medical Center (UMMC) in Kuala Lumpur, Malaysia from July 2008 till July 2012. Patient blood samples were confirmed for *P. knowlesi* malaria infection by several tests including microscopic examination, BinaxNOW malaria rapid diagnostic test (Inverness Medical International, Stockport, United Kingdom) and PCR based on the *Plasmodium* small subunit ribosomal RNA genes [15]. A control group of blood samples (n = 60) obtained from healthy donors was included in the study. The donors consists of ‘orang asli settlement samples as well as patient samples from UMMC hospital that were diagnosed as malaria negative. The ‘orang asli’ samples were taken randomly from various settlements around Malaysia. All samples had no previous malarial infections and all blood samples were screened by PCR.

Ethical approval for this study was obtained from the University of Malaya Medical Centre Ethic Committee (MEC Ref. No. 817.18) and informed verbal consent from the donor or the next of kin was obtained for use of these samples in diagnosis. Written consent was found to be unnecessary as verbal consent would be sufficient for the purpose of this study and patient details were noted down for our personal recordkeeping. This consent procedure was approved by the University of Malaya Medical Centre Ethic Committee.

### Genomic DNA extraction and ASP-PCR

Genomic DNA was extracted from blood samples using a commercial blood extraction kit following the manufacturer's protocol (QIAGEN, Hilden, Germany). The Duffy blood group genotypes were determined using ASP-PCR based on four sets of primers as previously described [19]. Briefly, samples of genomic DNA were subjected to three different PCR reactions containing a combination of the four primers described. Two of the PCR reactions contained the *FY* forward primer paired with individual FY*A and FY*B specific reverse primers, whereas, the last reaction used a combination of a forward primer that annealed to the mutated promoter region of the FY*B^ES^ and the FY*B specific reverse primer. All three reactions would yield a PCR product of 713 base pairs in length.

Reaction conditions were optimized to ensure specific amplification and these included primer concentration, Mg^2+^ concentration, genomic DNA template concentration and annealing temperature. Amplification was performed using approximately 0.5 µg of genomic DNA in a final volume of 20 µl which also contained 0.4 µM of forward and reverse primers, 0.2 mM of dNTP, 2 mM MgCl_2_ and 1 unit of Taq DNA polymerase in appropriate buffer (Promega, Madison, WI). PCR conditions were initiated with an initial denaturation of one cycle at 94°C for 2 minutes followed by 30 cycles of 30 seconds at 94°C, 1 minute at 60°C for annealing and 1 minute at 72°C performed in a Biorad MyCycler thermal cycler (Biorad, Hercules, CA). All PCR reactions were terminated after a 10 minute extension at 72°C and PCR products were analyzed by gel electrophoresis on a 2% agarose gel stained with SYBR Safe DNA gel stain (Invitrogen, Eugene, OR).

### Statistical analysis

Statistical analysis was done using Fischer's exact test in order to assess the significance of the genotypic and allelic variables in this study and to obtain independence among the proportions of Duffy genotype alleles in *P. knowlesi* infected patients and blood donors.

## Results

Duffy genotyping was successfully performed on 60 blood donor and 51 *P. knowlesi* infected patient samples by ASP-PCR. The proportion of predicted phenotypes of the Duffy blood group for each blood sample is summarized in Table 1. The data showed high frequency of the Fy(a+b−) phenotype, i.e., in 99 individuals (89.2%) of the total samples. Distribution of the corresponding FY*A/FY*A genotype in the blood donors and infected patients indicated this was the dominating genotype, at 85.0% and 94.1% respectively. No significant difference (P = 0.140) was observed between the donor and malaria infected patients for this genotype. The genotypic frequencies of the Duffy blood group for each blood sample are summarized in Table 2.

**Table 1:** Proportion of predicted phenotypes of blood donors and *P. knowlesi* infected patients.

Predicted phenotype	No. of individuals
**Fy (a+b+)**	10 (9.0%)
**Fy (a+b**−**)**	99 (89.2%)
**Fy (a**−**b+)**	2 (1.8%)
**Fy (a**−**b**−**)**	0

**Table 2:** Comparison of genotypic frequencies between blood donors and *P. knowlesi* infected patients.

Genotypes	Donors (n = 60)	Patients *P. knowlesi* (n = 51)	P-value
FY*A/FY*B	7 (11.7%)	3 (5.9%)	0.338
FY*A/FY*A	51 (85.0%)	48 (94.1%)	0.140
FY*A/FY*B^ES^	0	0	
FY*B/FY*B	2(3.3%)	0	0.499
FY*B/FY*B^ES^	0	0	
FY*B^ES^/FY*B^ES^	0	0	

Frequency of heterozygous genotypes in both groups was low, 7 in the blood donor and 3 in the infected patient groups, this being the FY*A/FY*B genotype. Again, there was no significant difference between the groups (P = 0.338) and with low frequency of the genotype, amounted to 9% of the total predicted phenotype of both groups. The FY*B homozygous genotypes was only observed in the blood donor group with distribution of 3.3%. The genotypes FY*A/FY*B^ES^, FY*B/FY*B^ES^, and FY*B^ES^/FY*B^ES^ were absent in the blood donors and infected patients. The phenotype Fy (a−b−) was therefore absent.

As for allelic distribution, which have been summarized in Table 3, the FY*A allele was found to be predominant in both, 90.8% for blood donors and 97.1% for *P. knowlesi* infected patients. The allelic frequencies of the FY*B allele, on the other hand, were found to be low, 9.2% in blood donors and 2.9% in infected patients The FY*B^ES^ allele was not detected in either blood group. The distributions of FY*A and FY*B alleles were not significantly different (P = 0.094) between both groups. This lack of difference between genotypic and allelic distribution between the blood donor group and the *P. knowlesi* infected patients seems to suggest that neither factor affects malarial infection susceptibility.

**Table 3:** Comparison of allelic frequencies between blood donors and *P. knowlesi* infected patients.

Alleles	Donors (n = 60)	Patients *P. knowlesi* (n = 51)	P-value
FY*A	109 (90.8%)	99 (97.1%)	0.094
FY*B	11 (9.2%)	3 (2.9%)	0.094
FY*B^ES^	0	0	

## Discussion

For the most part, natural resistance to *P.*vivax and *P.knowlesi* malaria infection in humans has largely been attributed to the Duffy blood group especially so in the case of individuals with the Fy(a− b−) or Duffy silent phenotype. Recently, exceptions to this resistance have been reported for *P. vivax* infections in South America especially in the Brazilian Amazon as well as Kenya and Madagascar [20], [21], [22], [23]. Duffy blood group polymorphisms are important in areas endemic for *P. vivax* and *P. knowlesi* infection as it provides a route for the parasite's entry into the erythrocyte. The data provided in this study emphasizes the importance of Duffy genotype in areas endemic for *P. knowlesi* infection.

Our results show that the FY*A allele has the highest frequency in both the blood donor and infected patient groups. This is expected as Oriental Asian populations have a propensity to have high frequency of this allele. Indeed, the Fy^a^ antigen is common among the Chinese, Japanese and Melanesians but not among Black Africans [24], [25]. The Fy^b^ antigen on the other hand, is found more frequently in Caucasian populations [26], [27]. None of our samples had the FY*B^ES^ allele nor the FY*B^ES^/FY*B^ES^ genotype and Fy (a−b−) phenotype. This is probably due to the low occurrence of the allele in this region as the FY*B^ES^ allele is generally found in those of Black African ancestry. Thus, without any Duffy negative samples we were unable to compare the susceptibility of Duffy positive and Duffy negative individuals to *P. knowlesi* infection in our study population.

However, it has been reported that other genotypes besides Duffy negative are capable of influencing susceptibility to *P. vivax* and possibly *P. knowlesi* infections [20], [28] This was not observed in our study as the genotype frequencies were not significantly different between blood donors and malaria infected patients, especially in the FY*A/FY*A genotype. High frequency of the said genotype however is in accordance with a study on the global Duffy blood group where the distribution of genotypes in the Asian continent was found to be dominantly FY*A/FY*A (96.86%) [29]. Also, the frequencies of the FY*A allele found in our study mirror those reported where the median frequencies were predicted to be above 80% across large extents of south Asia, Australia and in populations of Mongolia and eastern China and Russia. Our study also corroborates a prior study on the Duffy blood group distribution in Malaysia, where 99.68% ‘orang asli’ aborigines possessed the FY*A allele and no Duffy-negative individuals were detected. Our results indicate that the genotype distribution has not changed much in the country since the last study was conducted in 1988 [30].

In summary, the data obtained from this study reveal the genotypic distribution of the Duffy blood group among *P. knowlesi* infected patients in Peninsular Malaysia. Also, no significant difference was observed between frequencies of the preponderant FY*A/FY*A genotype between the control and malaria infected group. Nonetheless, our results need to be corroborated by further evaluation with a larger sample size as well as incorporating serotyping of the same samples coupled with ASP-PCR which would provide a better analysis of the genotypic distribution in the country as well as susceptibility of *P. knowlesi* malaria to particular Duffy genotypes.
